# Trajectories of severe eating disorders through pregnancy and early motherhood

**DOI:** 10.3389/fpsyt.2023.1323779

**Published:** 2024-01-05

**Authors:** Bente Sommerfeldt, Finn Skårderud, Ingela Lundin Kvalem, Kjersti Gulliksen, Arne Holte

**Affiliations:** ^1^Institute for Eating Disorders, Oslo, Norway; ^2^Department of Psychology, Faculty of Social Sciences, University of Oslo, Oslo, Norway; ^3^Faculty of Health Sciences, University of Southern Denmark, Odense, Denmark; ^4^Faculty of Health and Sport Sciences, University of Agder, Kristiansand, Norway; ^5^The Norwegian Psychological Association, Oslo, Norway; ^6^Norwegian Institute of Public Health, Oslo, Norway

**Keywords:** eating disorder, pregnancy, postpartum, protective factors, triggers, precursors

## Abstract

**Background:**

During pregnancy and early motherhood, risks of relapse and worsening are high for women with a history of eating disorders (EDs), as are adverse sequelae for their babies. However, systematic descriptions of the processes that these women undergo through pregnancy, birth, and early motherhood are lacking, as are good descriptions of the various trajectories these women follow through pregnancy and early motherhood. This study addresses both these knowledge gaps.

**Methods:**

We used a longitudinal research interview design, recruiting a non-clinical sample of 24 women with a history of severe EDs from routine pregnancy controls in five public, local, family health care centers in Norway. The participants were interviewed twice, first during pregnancy and then 4–6 months after delivery. Data were analyzed according to grounded theory. The focus was on modeling the trajectories of EDs through pregnancy, birth, and early motherhood. All the participants were diagnosed (DSM-5) using the Eating Disorder Examination and then completed the Eating Disorder Examination Questionnaire.

**Results:**

Five perceived trajectories through pregnancy and early motherhood were identified: “The mastering mother,” in which an ED pathology seems to be absent through pregnancy and early motherhood; “The inadequate mother,” in which the ED pathology worsens before pregnancy, through pregnancy, and early motherhood; “The overwhelmed mother,” in which the ED worsens during pregnancy and early motherhood; “The depressed mother,” in which the ED is put on hold during pregnancy, but worsens in early motherhood; and “The succeeding mother,” in which the ED worsens during pregnancy, but reduces in early motherhood.

**Discussion:**

ED trajectories through pregnancy and early motherhood vary greatly among women with a history of EDs. This may indicate different psychological dynamics through these phases. A model with five trajectories captures a large degree of the variation. The model may help clinicians’ preparedness when dealing with these patients.

## 1 Introduction

Eating disorders (EDs) encompass behaviors such as restrictive eating, fasting, binging, and self-induced vomiting, as well as psychological traits such as distorted cognition and preoccupation with weight, body image, body, and food (1). In both anorexia nervosa (AN) and bulimia nervosa (BN), self-worth relies on body, shape, and weight (2).

Pregnancy and early motherhood are known to be vulnerable times for women with EDs (3–7). Reported prevalence during pregnancy is 5–8% (8, 9), increasing to 12.8% after birth (9), compared with 0.8% for AN and 2.8% for BN for comparable age groups in the general population (10). One assumption is that these differences are linked to profound and rapid changes in body appearance, body image, body functions, body sensations, and sense of self-identity during these phases in life (5, 11–13).

The distorted cognitions and behaviors associated with EDs have been observed to persist through pregnancy and postpartum (14, 15). Women who experience persistent ED pathologies through pregnancy and early motherhood also tend to report higher levels of anxiety and depression (8, 16, 17). Furthermore, postpartum depression seems to occur more frequently in women who have or have had an ED (16, 18). Taborelli et al. (19) examined retrospectively the transition from pregnancy to motherhood among women with a current ED. Recurrence of ED symptoms and depressive symptoms after birth were experienced by the subjects as a sign of their inadequacy as a mother (19).

EDs during pregnancy and early motherhood also seem to be a potential risk factor for the fetus (20–22). For instance, EDs are associated with increased risk of problems during labor and delivery, including higher risk of premature birth and cesarean delivery (23, 24), fetal distress (17), and resuscitation, low Apgar scores in newborns, and perinatal death (25).

Despite all this, the intersection between the ED pathology, pregnancy, and motherhood is little studied. Most studies have used questionnaire data (22), were retrospective (19), and/or used clinical hospital samples (1, 19). Although important, such studies have significant limitations. Some are likely to suffer from recall bias, selection bias, or, as in some questionnaire studies, are likely to give only quantitative, epidemiologically useful, but relatively superficial answers of limited clinical value (15, 26, 27).

Moreover, the available studies are limited to only one particular period of motherhood: pregnancy, postpartum, or early motherhood. For instance, Tierney et al. (1) investigated the experience of pregnancy among women with an ED and described their conflicting loyalties to their ED and their future baby’s demands. The authors suggested that this conflict can cause women to question their mothering skills and deplete their already diminished self-esteem. Mason et al. (28) studied how women with AN have difficulty adapting to their new status and the emergence of a new relationship with their body during pregnancy. Others have found that women with EDs in the postpartum period report being highly distressed by breastfeeding and by the intensity of their body image and shape concerns (29).

No study has yet explored the psychological processes that these women go through or the possible different trajectories through pre-pregnancy, pregnancy, and early motherhood. Nor are there any prospective studies of the trajectories of ED pathologies through these phases of life.

Is it possible to identify a typical trajectory for such women? Are there some common traits, factors, or processes that the women themselves link to worsening, relapse, or maybe even improvement and recovery from their ED pathology through these phases? To treat this vulnerable group of women in a caring manner, it would be helpful to have a better understanding of how they experience the trajectory of their eating problems through pregnancy and early motherhood.

To start filling the gaps in the knowledge and provide clinicians with some insights, we used a longitudinal in-depth study design to closely follow a non-clinical sample of 24 women with a history of severe EDs through pregnancy and the first 6 months after birth. The participants were a non-clinical group, meaning that none of them were in treatment for their ED when they became pregnant and that they were recruited from a universal, public, routine pregnancy control, not a clinical treatment facility. Severity was conceptualized and defined using three components: persistent symptoms, long duration, and treatment history (more than 7 years). Weight was not used to determine severity because of the fluctuating weight of pregnant women. We aimed to identify possible common structural components in the women’s narratives, as well as possible differences.

We had two research questions: (1) How do women with a history of severe EDs experience their ED pathology during the process from pregnancy to postpartum and (2) On the basis of these experiences, is it possible to identify different trajectories through pregnancy and early motherhood?

## 2 Materials and methods

### 2.1 Participants

This study is part of a larger study, “Mummy bodies.” In “Mummy bodies,” we comprehensively interviewed 24 women twice, first during pregnancy and then 4–6 months after delivery. Inclusion criteria were that the participants had a self-reported history of an ED, had been in treatment for a diagnosed ED within the past 10 years, and were pregnant at the time of the first interview. Exclusion criteria were any psychotic symptoms.

### 2.2 Setting and procedure

The participants were recruited from the general population through the universal, public, routine pregnancy controls at five local family health care centers in the Greater Oslo area in Norway. The services are free. The participation rate in the pregnancy controls at these centers is 95–98% (30).

Midwives and nurses at the health care centers were thoroughly informed about the project and its inclusion and exclusion criteria before being asked to invite potential participants. Social media, podcasts, and seminars were used to encourage and support the recruitment of participants. We did not know the degree to which the sample was representative of pregnant women with a history of EDs; moreover, the study did not differentiate between women with a current history of EDs and women who had previously suffered from EDs. The Eating Disorder Examination (EDE) and Eating Disorder Examination Questionnaire (EDE-Q) were used to qualify patients’ current ED status.

Those interested in participating sent an email to the first author and then received further information about the study by email, accompanied by a written invitation to participate. The written invitation contained a detailed description of the research project, including its purposes and procedures. No one withdrew after having consented to give their contact information to the researcher.

After participation had been approved, the participant and interviewer arranged a time to meet. Then, the two semi-structured research interviews were conducted, with the participants completing the EDE-Q after each one (31). Further, the EDE was administered after each interview to make the ED diagnosis based on DSM-5 (32). All the information about the participants’ ED history was based on self-reporting.

The aim of the interviews was to provide rich descriptions as precise and as close to the participants’ experiences as possible. Data were collected using the “Experience Interview” technique (33), a semi-structured participant-centered, strategic conversation format developed from communication theory (34).

All the interviews were conducted by the first author (BS). Each interview lasted between 90 and 120 min and was audiotaped and transcribed verbatim by the same author. Altogether, providing the introductory information, conducting the interviews, and administrating the EDE-Q and EDE took two and a half hours over the two meetings, a total of 5 h for each participant. All the procedures were conducted in accordance with the Helsinki Declaration, and the study was approved by the Norwegian Regional Committee for Medical Research Ethics.

### 2.3 Data analyses

We used guidelines from grounded theory to develop an empirical model of trajectories based on the data collection and analyses of the women’s experiences (35). To analyze the interviews, NVivo 12 was used to organize the data (36). Subsequently, summaries and notes from the transcription process were analyzed. The analytical process was not carried out in isolation; rather, it was based on immersion in the data and repeated sortings, codings, and comparisons as per grounded theory (37). Ensuring a continuity of descriptions and interpretations was important throughout the process.

The analyses involved seven main steps. First, the first author, an experienced clinical psychologist and psychotherapist specialized in EDs, conducted the interviews; further familiarization with each narrative was achieved by listening to the audio files, transcribing the interviews verbatim, checking the accuracy of the transcripts, and reading and re-reading the transcripts. Concurrently, the second (FS) and fifth (AH) authors listened to the tape recordings and participated as co-readers of the transcripts and as discussants of possible interpretations. The first author made detailed notes from the interviews as well as observations and comments about potential significance to gain an overall impression and capture relevant and varied experiences. Throughout the process, we reflected upon potential biases and preconceived ideas that we might have brought to the analyses and that could possibly influence our interpretations.

The second step involved analyzing the interview transcripts. Each text was examined using open thematic coding (35) according to the bottom-up principle (38, 39) to reveal different themes. We examined the individual words, phrases, and sentences in each transcript and identified relevant categories. During the discussion of the interviews, the team became aware of some tendencies associated with being pregnant and a mother and the emergence of symptoms of EDs. All the text excerpts that included statements about ED symptoms through pregnancy and early motherhood as well as about being a mother were coded and labeled according to their meaning.

Third, we explored different outcomes from the transcribed interviews from the postpartum period. We then looked back on the birth and pregnancy descriptions to see how different trajectories led to different outcomes. We summarized each woman’s experiences of ways in and out of eating problems during pregnancy and postpartum. Each summary was used to identify experiences and how their thoughts and practices relating to their body, weight, and food through pregnancy and early motherhood emerged.

In the fourth step, we identified possible common structural components in the women’s experiences of the trajectory of their ED pathology. We systematized the experiences that showed different trajectories through open coding and validation against the original text using confirmatory and selective coding (35). This resulted in three higher order constructs: precursors, triggers, and protective factors.

Fifth, we conducted axial coding (35). While open coding refers to the deconstruction of data (i.e., breaking data apart), axial coding refers to the reconstruction of data. In this process, we explored how the different higher-order constructs were related to each other and grasped the complexity of the women’s narratives. This allowed us to describe the experiences longitudinally from before pregnancy to birth and early motherhood. In this part of the analyses, experiences from the higher-order constructs were combined into five trajectory constructs, which represented the lowest number of constructs needed to describe how the participants perceived their trajectory to motherhood. The language of the participants guided the trajectories’ labels. Their experiences were identified with short descriptions, which became the name of the trajectory. The five trajectory constructs were labeled “The mastering mother,” “The succeeding mother,” “The inadequate mother,” “The depressed mother,” and “The overwhelmed mother.” This coding provided us with a model that accommodated the individual variations in the participants’ narratives in terms of precursors, triggers, and protective factors.

Sixth, to ensure that each trajectory fitted with the original narratives, the data were analyzed a third time according to the top-down principle. Through this process, the constructed trajectories were validated by checking each pathway against the original texts to determine whether further refinements were needed to adequately represent the original data and minimize overlaps between the trajectories.

Finally, to check credibility, the analyses were regularly discussed within the research team to ensure that the themes were well represented in the data and vice versa. As a result, the themes and interpretations were continuously challenged, discussed, and reassessed.

## 3 Results

### 3.1 Participants

Earlier diagnosis is self-reported based on the DSM-IV diagnosis. Years of treatment are self-reported. Diagnosis is based on the DSM-5 criteria. Postpartum depression is diagnosed by a GP, and self-reported by the participants to the interviewer. The participating women were between 26 and 42 years of age (mean 32.8 years). The average age of first-time mothers in Norway is 30.6 years (2021). All the participants reported having been in treatment for a diagnosed ED within the preceding 10 years, with a duration of treatment for their ED ranging between 2 and 17 years (mean 7.7 years). None were in specialized treatment for their ED when they became pregnant.

A total of 22 of the 24 women said that they had needed treatment again during pregnancy. Nine of the participants restarted specialized treatment for EDs while they were pregnant, 13 reported that they had asked for treatment for their eating problems during pregnancy at the birth controls but did not receive specialized treatment, and two did not need specialized treatment during pregnancy. There were 13 first-time mothers, nine second-time mothers, and two third-time mothers. Seven of the participants became pregnant through *in vitro* fertilization (IVF). Gestation week at the time of the interview ranged from week 9 to week 40. Week of birth ranged from week 35 to week 40 (mean week 38.8).

Altogether, 23 of the 24 participants received an EDE-assessed DSM-5 diagnosis of an ED at the time of the first interview, while 21 received an EDE-assessed DSM-5 diagnosis of an ED at the time of the second interview. During pregnancy, three were shown to have BN, two were shown to have an unspecified feeding or eating disorder (UFED), and 18 were shown to have a type of other specified feeding or eating disorder (OSFED). At postpartum, two were shown to have BN, six were shown to have an UFED, and five were shown to have an OSFED. Few women met the full DSM-5 criteria for an ED diagnosis due to their pregnancy weight. The symptom pressure measured by the EDE-Q Global Score was generally high. The mean EDE-Q Global Score for the 24 participants was 3.54 during pregnancy and 3.41 at postpartum. Finally, postpartum depression was diagnosed by the medical general practitioner in 14 of the 24 women. Breastfeeding was performed by 20 of the 24 women.

### 3.2 Structural components

The comprehensive analyses of the 24 women identified three common structural components: precursors, triggers, and protective factors. The three components were all addressed in all of the 24 women’s narratives.

Precursors comprised conditions such as ED history, number of births, personal characteristics, family history, body image, family transmission, and/or attitude toward pregnancy. These experiences were present before the women became pregnant and were regarded by the participants as important to their way of dealing with pregnancy and early motherhood.

Triggers were distinctive events that the participants linked to relapse or worsening of their EDs during their pregnancy, birth, or early motherhood, such as an unplanned pregnancy, ambivalence to pregnancy, IVF, change in appearance, body image, negative comments about their body, and/or lack of support.

Protective factors were experiences and events the participants linked to positive ways of dealing with pregnancy and early motherhood and that they associated with improvements in their ED pathology, such as positive body experiences, small body changes, a sense of achievement, a sense of mastery, and/or relationship satisfaction/support.

### 3.3 ED trajectories

On the basis of the combinations of the various content themes within the common structural components described above, we constructed five trajectories of perceived relapse, worsening, improvement, and/or recovery of the ED pathology during pregnancy and early motherhood (Table 1). In the subsections below, each trajectory is illustrated by condensed quotations from three or more participants that led to the inference of that particular path. Each trajectory is based on the mother’s own statements about the experiences of the ED pathology during pregnancy and at postpartum. Information that could reveal a participant’s identity has been removed.

**Table 1:** Five trajectories of EDs through pregnancy and early motherhood.

1. “The mastering mother”: ED pathology seems to be absent through pregnancy and early motherhood.
2. “The succeeding mother”: ED worsens during pregnancy but reduces in early motherhood.
3. “The inadequate mother”: ED worsens before pregnancy, through pregnancy, and early motherhood.
4. “The depressed mother”: ED pathology is put on hold during pregnancy but worsens in early motherhood.
5. “The overwhelmed mother”: ED worsens during pregnancy and early motherhood.

#### 3.3.1 The mastering mother: recovering through pregnancy and early motherhood

This trajectory is based on three of the participants. For the mastering mother, the role of being a mother is an important turning point in the trajectory of an ED. The narratives behind this trajectory are characterized by a woman experiencing being a mother as a clear task filled with mastery and a new meaning.

Precursors: Lower body mass index before pregnancy. Strong awareness of food intake and exercise were present in all the women, along with descriptions that were performance oriented and related to having a strong will. “*I have always been a person who sets goals and is obsessed about reaching them. I got obsessed with getting pregnant*.”

Women who followed this trajectory had a history of AN. They wanted to become pregnant. They planned their pregnancy, became pregnant quickly, and experienced having become pregnant as a great achievement. “*I became pregnant very quickly, and being pregnant is something I manage*.” They were also determined that their own relation to food, body, and weight should not affect their baby. This was a strong motivation for improvement.

Triggers: The narratives of these mothers contained hardly any references to distinct events that could be considered triggers for the ED pathology.

Protective factors: The women in the mastering mother category reported several protective factors. They experienced small body changes during pregnancy. “*I really enjoyed my small stomach during pregnancy. I was proud of it!*” They experienced good support and the pregnancy as a common project. “*This was our project. And I really enjoyed my husband touching my belly*.”

The protective factors continued through to delivery. The women delivered close to their delivery date. This gave them a feeling of safety. Most procedures and activities went as planned, and the mothers experienced a normal delivery. They also reported a sense of control over their body. “*I had a normal birth, and for the first time felt in control of my own body. It was stronger than I thought*.” “*Being able to feel pain and at the same time have a sense of control was important to me*.”

As mothers, they were obsessed with doing everything right, an obsession that functioned as a trigger, as in the trajectories of the succeeding mother and the depressed mother. Instead, they experienced mastery by being in good shape; this sense of mastery made them less stressed about thoughts about their body and food. “*Being a mother gives me a bit of a break from all these thoughts about food and exercise, and now this exercise and awareness of what I can eat finally makes sense. After all, now I have to keep myself healthy and fit for another person*.”

The feeling of mastery and achievement persisted. “*I lost weight right away, and that gave me a boost*.” They mastered breastfeeding, with the baby responding well and calming down easily. The women reported bonding well with the baby. “*I love his smile when he sees and hears me. It makes me forget everything else*.” “*This pregnancy and being a mother have meant I do not need the ED anymore. I am mastering something else now*.”

#### 3.3.2 The succeeding mother: improvement after birth

This trajectory is based on four of the participants. The ED became worse during pregnancy but subsided when the participants became mothers. Birth and early motherhood are important turning points for the trajectory of the ED in the succeeding mother. The narratives of this trajectory are characterized by how these mothers struggled during early pregnancy but experienced birth and being a mother as something that gave them a feeling of being someone. They grew into the role of being a mother.

Precursors: Before becoming pregnant, these mothers had a history of AN with a combination of low self-esteem, feeling not being good enough, and perfectionism. Their ED had been a way of giving them a sense of succeeding at something. “*I have never been satisfied with something halfway, and I wanted to be the perfect pregnant and the perfect mom. I think I mean thin and healthy*.” To these women, pregnancy became difficult to deal with alongside their precursors of rigidity and inflexibility. “*I have always been a person who had plans and rules for everything. Just like I planned my pregnancy*.”

Triggers: The ED triggers of the succeeding mother kicked in at the first trimester if they had become pregnant earlier than planned, had strong fears about becoming a mother, and had concerns about the changes to their body beyond their control. “*I need to have control over everything I do, but when I got pregnant nothing went as I had planned*.” Women following this trajectory seek to ensure a thin pregnancy, with strict rules and fixed regimes. This focus makes it difficult to connect to the baby during the first two trimesters.

Protective factors: Protective factors in the succeeders arose if they had a small stomach and gained less weight than they had feared. The worsening of their ED pathology eased and even declined when these mothers were able to connect to their baby through its activity and movements. The mothers enjoyed sharing these moments with their partner. In the last trimester, these women even allowed themselves to dream of becoming a good mother.

The mothers in this trajectory may experience protective factors through delivery if the birth is planned by cesarean section. This makes the delivery predictable and gives the women more of a sense of control. In our study, all four succeeding women experienced a good sense of control over their delivery.

This feeling of control continued through early motherhood. After delivery and in early motherhood, in this trajectory the triggers became less dominant, which led to improvements in the ED pathology that had worsened during pregnancy. Being a mother was something that succeeders managed and that gave them a feeling of self-confidence. “*The role of being a mother gave me a focus and a clear role*.” “*In a way, I avoided walking around feeling inept, as I had done in all other areas*.” This led to less fear about not being good enough. Through the baby, the women obtained a sense of being “good enough.” Their lack of self-confidence weakened when the contact with the baby was good. “*I am experiencing a mutual beneficial interaction and think I am valuable*.”

Those in the succeeding mother category felt good when they lost weight and when their bodyweight was back after a few weeks to what it was before pregnancy. Hence, one protective factor seems to be a feeling of contentment with their own bodies.

The mothers following this trajectory mainly experienced breastfeeding as unproblematic. Breastfeeding also seemed to contribute to healthier eating patterns. “*As long as I breastfeed, it is easier to eat regularly and be more flexible*.” In addition, a good collaborative relationship with their partner contributed to the positive development in this trajectory. “*My husband has been very supportive to me and gave me a lot of positive comments on me being a good mother*.” So, too, did simply being with the baby. “*My daughter makes me feel good. She makes me feel needed. It is like I no longer need the ED to feel special*.”

#### 3.3.3 The inadequate mother: inadequacies throughout pregnancy and early motherhood

This trajectory is based on seven of the participants. The women in the inadequate mother category reported that their ED pathology re-emerged or intensified before they became pregnant and continued to intensify through pregnancy and early motherhood. They interpreted their ED as something to hold onto when everything else was unpredictable. Throughout the trajectory, a feeling of insecurity dominated and intensified as the pregnancy developed to the time around delivery and beyond.

Precursors: All seven women in this trajectory had a history of severe AN. Several of them had suffered from anxiety in their childhood. “*I was always described as a very worried type through my childhood. Now, worrying about another person is making this worse*.” These women came to pregnancy with a general feeling of insecurity and a high level of anxiety. Even before becoming pregnant, they were scared of doing something wrong that could harm the baby in the future. Most of them were therefore ambivalent about becoming pregnant, and when they became pregnant, the pregnancy was difficult to deal with.

Triggers: Six of the seven inadequate mothers became pregnant through IVF. For all of them, the inability to become pregnant and difficulties in becoming pregnant through IVF were a trigger for their ED, which developed further into pregnancy. They experienced their inability to become pregnant naturally as a loss of control. “*In the past, the ED was a lot about mastery and control. I have set myself goals and managed to reach them. When I wasn’t able to get pregnant, I had no control*.”

All the women in this trajectory experienced pregnancy as extremely overwhelming and as something that triggered feelings of guilt and self-contempt. “*I’ve ruined myself all these years with my ED. It’s my fault. And now I cannot focus on the baby, but only myself. What kind of a mother will I be?*” Throughout pregnancy, these mothers set high expectations for themselves and how they might appear as mothers. “*I have always been concerned about what others think of me. What did other people think of me, knowing about my history with EDs? This was something I was obsessed about during pregnancy and as a mother*.” Together with the continued fear of doing something wrong that could harm the baby, these expectations seemed to nourish the ED pathology.

All the brooding and rumination over doing things right led to the mothers feeling detached from their body and their baby. They were not sensitive to the baby’s movements and were afraid that they would not notice if something was wrong. During pregnancy, they had a strong focus on excessive exercise and made detailed plans to “*get the body back*” after birth. The IVF process also triggered disgust toward their own body that lasted throughout the pregnancy and after birth. “*My body is bulky and disgusting. I do not recognize it*.”

The feeling of inadequacy during pregnancy continued into feeling inadequate as a mother. Excessive exercise continued to take their attention away from their baby. They hoped that by focusing on their body shape, appearance, and weight, they would feel better about themselves. This focus helped them obtain a sense of mastery but reduced their attention on their baby. Their parenting thus became inconsistent and unpredictable. “*I cannot focus on the child right after eating. I become completely distant*.”

At the same time, they were afraid that they would no longer enjoy time with their baby. “*It takes up too much of my time, and there is none left to focus on the child*.” Overconcern for how others might think of them as a mother led these mothers to retain their strict ED regimes. They did not dare prioritize activities other than the baby and exercise. These mothers admitted that they found it difficult to differentiate between their own needs and their baby’s.

The women in the inadequate mother category felt that the quality of their motherhood was being judged. “*When I go to the controls with my baby, I feel like I am going to take an exam each time. It feels uncomfortable to put the baby on the weighing scales*.”

Feeding their baby poses a challenge for those with EDs. The baby’s appetite and hunger are difficult to deal with. “*I do not know whether she is hungry or just in need of comfort. I always offer breastfeeding first*.” Breastfeeding also became a way of losing weight or being able to eat “normal” amounts of food. “*Breastfeeding gives me permission to eat more than I normally can*.” Transitioning to solid food is difficult, and the fear of stopping breastfeeding is seen as a trigger. “*I then have to change routines, and that’s scary*.”

Those in the inadequate mother category struggle with feelings of not being a good enough mother. “*That someone else is completely dependent on me scares the crap out of me.*” Often, the mothers in the study did not feel enough support from their parents, husband, or friends. They felt a fear of being judged by others and were oversensitive to being negatively evaluated. “*I need to know I am doing things right. I need to hear it from both my partner and the health center*.”

Their ED pathology was something that the inadequate mothers held onto; strict routines and rules created a feeling of safety. “*The focus on the ED made me feel safer. I had something concrete to hold onto when everything else was unpredictable*.”

Protective factors: The narratives behind this trajectory contain only modest references to distinct events that could be considered factors protective against EDs.

#### 3.3.4 The depressed mother: EDs worsening after birth

This trajectory is based on ten of the participants. All were second- or third-time mothers. All these of the participants were diagnosed with postpartum depression after birth by a medical general practitioner. During pregnancy, the women in the depressed mother group put their ED pathology on hold by planning how to deal with body, weight, and shape changes after birth. The ED pathology intensified into early motherhood when nothing went as planned.

Precursors: These women noted that their body was changing in ways that differed from the first time they became pregnant. In our study, these women all had a history of BN. The depressed mothers referred to their ED pathology as involving the desire to hold onto still being identified as “*the thin girl*.” They often grew up with a mother who binged and purged and talked about how they gained weight through their pregnancies.

Triggers: During pregnancy, the women in this trajectory tended to experience rapid body changes. Detachment from their body and pregnant stomach triggered a fear of reawakening their ED pathology. These women made a rule for themselves that their ED behavior would not be something to fall back into, and they put all such behavior on hold during their pregnancy.

Among the depressed mother group, the delivery became a further trigger for their ED pathology. Nothing went as planned. In our study, all the women in this trajectory experienced complications. “*It all became very acute. Nothing went as planned, and it was so chaotic. I was supposed to have a normal birth, but then suddenly the situation went out of control. A lot of doctors and nurses were around me, my partner was gone, and everything happened very fast*.”

Several things worsened in the postpartum period. All the women in the depressed mother group were diagnosed with postpartum depression. They were not able to fulfill their plans about exercise and healthy dieting. This made them feel like a failure. “*I am nothing. I wasn’t able to complete anything. I got restless and exhausted*.” They had trouble soothing their baby. “*If he cried, nothing I did calmed him down. He was struggling with a lot of stomach difficulties and cried a lot*.” Breastfeeding was complicated and they could not manage it. “*I gave up. I was useless*.” Being the mother of two could be challenging. “*The ED was a way of not dealing with the challenges and my secret escape from all that I cannot deal with*.”

Protective factors: The women had managed to get their body back after the previous birth and the plan for how to achieve it this time protected them from ED behavior during pregnancy. This trajectory does not contain references to distinct events that could be considered protective factors against EDs after birth.

#### 3.3.5 The overwhelmed mother: chaos, shame, and guilt throughout pregnancy and early motherhood

This trajectory is based on six of the participants. The narratives behind this trajectory were characterized by using the ED pathology to deal with the chaos during pregnancy and early motherhood, as well as a kind of self-affirmation of “me as a bad person.” An ED pathology while being a mother increases self-blame and provokes shame and guilt.

Precursors: The women in this trajectory typically had a history of intense self-hatred that they linked to traumas in early childhood. These women regarded the ED as a way of dealing with chaos earlier in life. All the women in this trajectory had a history of BN and descriptions of impulsivity. “*I have always been described as impatient and as making decisions without thinking. That’s how this ‘pregnancy’ thing was seen too*.” They said that thanks to their treatment history, they had managed to bring more stability into their lives with routines and exercises. In this way, they had managed their impulsivity and BN in recent years.

Triggers: These mothers typically experienced pregnancy as a threat to their sense of control over their lives. They lost all their routines. “*It’s all so chaotic. Nothing works. I have lost my own way of living*.” Anger and self-hatred were other triggers. “*I feel so angry at this ‘thing’ inside me, which destroys my body and forces me to eat. And at the same time, I feel like a terrible person for feeling this way*.”

Difficulties in dealing with being pregnant triggered a worsening of the ED pathology. Through the ED pathology, they tried to disconnect from being pregnant. “*I cannot deal with the baby inside me. I cannot do it because it makes me feel guilty and like a terrible person for still doing all these harmful things to my body*.”

The worsening of the ED involved a vicious circle of purging and overeating. “*I need something that I can hold onto and help me to forget everything else. I cannot control anything right now. Planning my overeating helps me*.” The vicious circle continued into early motherhood. The ED became a protection for dealing with shame and guilt. “*Binging and purging allow me to detach from everything. It’s like a protection for me*.” “*I am afraid of disclosing this to the health care professionals. I am not able to stop, and I am afraid they will think I am a terrible mother*.”

In early motherhood, they expressed a fear of harming their child. These mothers were typically worried about transferring their problems to their babies. “*I am terrified that I am going to ruin my baby with my eating problems*.”

Three of these mothers delivered later than expected, resulting in a bigger body. They experienced a loss of control during the birth and found it difficult. This resulted in a feeling of being overwhelmed and an alien self. “*I lost all control during birth. My body was huge, and I did not recognize myself at all*.”

The hatred of their bodies triggered a further ED pathology. “*My body feels like an alien. I can still feel anger toward the baby for having destroyed my body*.” During pregnancy, they felt stuck in a body they hated. Because of the baby, they had lost control over their body. “*I hate this. I am stuck. No freedom*.” Members of the overwhelmed mother group typically expressed frustration with their maternal body as well. “*As a pregnant woman, I am like a container that needs to be burst so that the contents can come out. When you have sacrificed your body by giving birth, you must sacrifice it again by being bound to it 24/7. I simply hate it*.”

The disgust with themselves was very strong, making it difficult to form a relationship with their baby. “*I get terrified if she looks like me. I heard she had inherited my thighs. That made me feel really bad for her. Poor girl. I am hurting her*.” They experienced a sense of ambivalence toward their baby and a fear about connecting to the child. “*In one moment, I can feel intense love for him, but then I start to panic and search for reasons why I cannot allow myself this love. I cannot allow myself to think of myself as a mother and for her to feel love for me. Who do I think I am then?*” Some saw themselves in the baby and consequently could not find the baby lovable. That scared them.

For these mothers, breastfeeding became overwhelming. In the narratives, these women described breastfeeding as something “*suffocating*” and regarded their “*own milk as dirt*.” “*Despite the pain, I breastfeed 24/7. And I think I have to in order to be a good mother. I have no other way of calming the baby down*.” After some months, they decided to start with a bottle, and their partners took responsibility for the feeding. All of the women in this trajectory stopped breastfeeding.

Protective factors: Good support from the partner was identified as an important protective factor in early motherhood.

## 4 Discussion

In this study, we explored the experiences that women with a history of EDs go through from pregnancy to early motherhood. We aimed to describe this process from the women’s perspectives. On the basis of analyses of the women’s descriptions, we identified different trajectories of the ED pathologies during pregnancy, birth, and postpartum. To the best of our knowledge, this is the first study to examine these processes and trajectories among this highly vulnerable group of mothers.

It is widely accepted that pregnancy and postpartum are particularly vulnerable times for women’s mental wellbeing (40–42). However, in-depth descriptions of how women with a history of EDs experience the trajectory of their eating problems through these phases of life are lacking. Such descriptions are important because they can increase health care providers’ sensitivity to and understanding of differences in psychological dynamics between women with similar symptoms.

The results revealed great variations in the ED trajectories and in the experience of becoming a mother among these women. Some seemed to improve or recover from their ED pathology, whereas others relapsed or saw their ED intensify.

We identified five distinct trajectories into motherhood: the mastering mother, the succeeding mother, the inadequate mother, the depressed mother, and the overwhelmed mother. These five trajectories reflect the complexity of the ED pathologies during pregnancy and early motherhood. They show how women link combinations and varying degrees and qualities of precursors, triggers, and protective factors to improvement, worsening, or relapse through these phases of life.

In three of the trajectories, we found triggers for worsening or relapse during pregnancy that continued into early motherhood. In the depressed mother trajectory, the women seemed to put their ED pathology on hold during pregnancy, but it intensified after birth and into early motherhood. During those periods, they were unable to complete their plans and experienced failure as a mother. In the inadequate mother trajectory, the women seemed to be overwhelmed by anxiety and unpredictability that worsened their ED pathology before pregnancy when they failed to become pregnant. In the overwhelmed mother trajectory, it seemed to be the actual pregnancy that triggered the reappearance of the symptoms. In this trajectory, the women deviated significantly from those in the other trajectories. This trajectory included precursors of self-hatred directed toward themself as a whole, their body, and/or their baby.

These findings agree well with previous findings that pregnancy and early motherhood may be vulnerable periods for women’s mental wellbeing (41, 43) and can be an extremely challenging time for women with an ED (3, 26, 27, 29, 44, 45). They are also consistent with previous findings that women who experience anxiety, depression, and high body dissatisfaction during pregnancy are more likely to develop postpartum depression (46, 47).

In two of the trajectories we identified, the mastering mother and the succeeding mother, the process of becoming a mother seemed to lead to improvement and recovery. Underlying mechanisms seemed to be a sense of mastery, a new meaning in life, and a feeling of self-worth. Even though the women in both the mastering mother and the succeeding mother trajectories recovered in the postpartum phase, these trajectories appeared to differ during pregnancy. In the succeeding mother trajectory, the women experienced a relapse during pregnancy followed by improvement in early motherhood. These women experienced motherhood as something that gave them a feeling of being someone. This is consistent with previous findings that being a mother can change women’s sense of self-identity in a positive way and thereby reduce their ED pathology (14, 15, 17, 19). However, in the mastering mother trajectory, the improvement occurred during pregnancy and early motherhood, whereas in the succeeding mother trajectory, it occurred only in early motherhood. We also found that precursors such as perfectionism, rigidity, and high achievement may be protective factors associated with improvement. It is well known that being pregnant and becoming a mother affects a woman’s self-identity (42). This study suggests that in women with a history of EDs, becoming a mother is a determinant of relapse, worsening, or recovery from EDs.

We did not investigate the clinical implications directly, but we can assume that women in the mastering mother trajectory would profit from support that strengthens the positive focus on the role of being a mother. Women in the succeeding mother trajectory might need comfort, predictability, and help in making good routines for themselves and their baby. Women in the inadequate mother trajectory would need support to feel good enough, to be reassured that the baby is doing well, and to cope with emotional experiences. Women in the depressed mother trajectory may need help establishing routines to which they are able to stick. Women in the overwhelmed mother trajectory would most likely profit from help that emphasizes building self-compassion and reducing shame.

It should also be borne in mind that these five trajectories are not mutually exclusive. Despite the different combinations of structural components, the narratives of the participating women had several themes in common. These included low self-esteem, feelings of shame, and problems with emotional regulation. To translate the findings of this study into health care service procedures and treatment strategies, further research is needed. Most importantly, however, this study points to the need to take the patient’s experience into consideration when a woman with a history of EDs becomes pregnant or plans to become a mother.

### 4.1 Strengths and limitations

Many patients do not disclose their eating problems to health care providers. The strengths of this study are the relatively large number comprising the non-clinical sample of women with a history of severe EDs, the high-quality in-depth interviews, and the longitudinal design, with data collections during pregnancy and 4–6 months postpartum, including retrospective data from the delivery. The use of standard diagnostic interviews both during pregnancy and at postpartum strengthened the diagnostic validity of the study. Another strength is the inclusion of the categories of AN, BN, OSFED, and UFED in both data collections; most studies are limited to AN and BN at only one time point. By including OSFEDs and UFEDs, one might capture cases that still have an ED (48), but who do not fulfill all the criteria for a specific ED. Both OSFEDs and UFEDs might be more suitable to select pregnant women whose clinical characteristics can be temporarily masked during pregnancy. Studies show that OSFEDs and UFEDs may be as severe and longstanding as a specific ED (49).

This study has several limitations. All the participants had received some kind of psychotherapy for their ED. This may have increased their understanding of their condition that would not otherwise have been there. We did not know how representative the participants are of women with a history of EDs. Even though the recruitment setting was non-clinical, we cannot exclude the possibility that the recruitment process facilitated the recruitment of women with the most salient eating problems. Although the participants were diagnosed as having an ED, we did not know how many or who had recovered before they became pregnant. Past history of the ED and its severity were self-reported; the participants’ health records were not verified. Consequently, we could not differentiate between those women who had an ED diagnosis when they fell pregnant and then worsened and those who had recovered and then relapsed. Additionally, having the same person conduct the semi-structured interviews and EDE might have influenced the responses in the interviews and the outcomes of the diagnosis. The interview time point during pregnancy varied between week 9 and week 40 and at postpartum between 4 and 6 months after delivery. Finally, we did not include the participants’ partners. They might have provided us with comparative information.

## Data availability statement

The original contributions presented in the study are included in the article/supplementary material, further inquiries can be directed to the corresponding author.

## Ethics statement

The studies involving humans were approved by Regional Committees for Medical and Health Research Ethics (REC) 20th of May 2020, Reference 92665. The studies were conducted in accordance with the local legislation and institutional requirements. Written informed consent for participation in this study was provided by the participants’ legal guardians/next of kin. Written informed consent was obtained from the individual(s) for the publication of any potentially identifiable images or data included in this article.

## Author contributions

BS: Conceptualization, Data curation, Formal analysis, Methodology, Project administration, Resources, Validation, Writing – original draft, Writing – review & editing. FS: Formal analysis, Methodology, Project administration, Supervision, Validation, Writing – review & editing. IK: Project administration, Validation, Writing – review & editing. KG: Methodology, Project administration, Supervision, Validation, Writing – review & editing. AH: Conceptualization, Data curation, Formal analysis, Methodology, Project administration, Supervision, Validation, Writing – review & editing.
