# Corrigendum: Trajectories of severe eating disorders through pregnancy and early motherhood

**DOI:** 10.3389/fpsyt.2025.1601996

**Published:** 2025-06-10

**Authors:** Bente Sommerfeldt, Finn Skårderud, Ingela Lundin Kvalem, Kjersti Gulliksen, Arne Holte

**Affiliations:** ^1^ Institute for Eating Disorders, Oslo, Norway; ^2^ Department of Psychology, Faculty of Social Sciences, University of Oslo, Oslo, Norway; ^3^ Faculty of Health Sciences, University of Southern Denmark, Odense, Denmark; ^4^ Faculty of Health and Sport Sciences, University of Agder, Kristiansand, Norway; ^5^ The Norwegian Psychological Association, Oslo, Norway; ^6^ Norwegian Institute of Public Health, Oslo, Norway

**Keywords:** eating disorder, pregnancy, postpartum, protective factors, triggers, precursors

In the published article, there was an error in **Table 1** as published. There are concerns of backwards identification risk. **Table 1** has been removed from the article. The descriptions have been retained as below.

A correction has been made to **3 Results, *3.1 Participants*, paragraph 1.**

This sentence previously stated: “Background information, diagnoses, and a brief history of the participants’ EDs are shown in **Table 1** for both interviews.”

The sentence was removed. The below sentences were added:

“Earlier diagnosis is self-reported based on the DSM-IV diagnosis. Years of treatment are self-reported. Diagnosis is based on the DSM-5 criteria. Postpartum depression is diagnosed by a GP, and self-reported by the participants to the interviewer.”

A correction has been made to **3 Results, *3.3 ED trajectories*, paragraph 1.**

This sentence previously stated:

“In the subsections below, each trajectory is illustrated by condensed quotations from one or three or more participants that led to the inference of that particular path.”

The corrected sentence appears below:

“In the subsections below, each trajectory is illustrated by condensed quotations from three or more participants that led to the inference of that particular path.”

A correction has been made to the numbering of the Tables in **3 Results, *3.2 Structural components*.**

This sentence previously stated:

“Table 2 Five trajectories of EDs through pregnancy and early motherhood.”

The corrected sentence appears below:

“Table 1 Five trajectories of EDs through pregnancy and early motherhood.”

A correction has been made to **3 Results, *3.1 Participants*, paragraph 3.**

This paragraph previously stated:

“Altogether, 23 of the 24 participants received an EDE-assessed DSM-5 diagnosis of an ED at the time of the first interview, while 21 received an EDE-assessed DSM-5 diagnosis of an ED at the time of the second interview (**Table 1**). During pregnancy, three were shown to have BN, two were shown to have an unspecified feeding or eating disorder (UFED), and 18 were shown to have a type of other specified feeding or eating disorder (OSFED). At postpartum, two were shown to have BN, six were shown to have an UFED, and five were shown to have an OSFED (**Table 1**). Few women met the full DSM-5 criteria for an ED diagnosis due to their pregnancy weight. **Table 1** also shows that the symptom pressure measured by the EDE-Q Global Score was generally high.”

The corrected paragraph appears below:

“Altogether, 23 of the 24 participants received an EDE-assessed DSM-5 diagnosis of an ED at the time of the first interview, while 21 received an EDE-assessed DSM-5 diagnosis of an ED at the time of the second interview. During pregnancy, three were shown to have BN, two were shown to have an unspecified feeding or eating disorder (UFED), and 18 were shown to have a type of other specified feeding or eating disorder (OSFED). At postpartum, two were shown to have BN, six were shown to have an UFED, and five were shown to have an OSFED. Few women met the full DSM-5 criteria for an ED diagnosis due to their pregnancy weight. The symptom pressure measured by the EDE-Q Global Score was generally high.”

A correction has been made to **3 Results, *3.3 ED trajectories*, paragraph 1.**

This sentence previously stated:

“On the basis of the combinations of the various content themes within the common structural components described above, we constructed five trajectories of perceived relapse, worsening, improvement, and/or recovery of the ED pathology during pregnancy and early motherhood (**Table 2**).”

The corrected sentence appears below:

“On the basis of the combinations of the various content themes within the common structural components described above, we constructed five trajectories of perceived relapse, worsening, improvement, and/or recovery of the ED pathology during pregnancy and early motherhood (**Table 1**).”

In the published article, there was an error in **3 Results, *3.1 Participants*, paragraph 2**. The sentence which includes the number of IVF is wrong.

This sentence previously stated: “Four of the participants gave birth through cesarean section”

The sentence was removed.

The authors apologize for these errors and state that this does not change the scientific conclusions of the article in any way. The original article has been updated.

